# Enhanced Survival of 22–25 Week Preterm Infants After Proactive Care Implementation: A Comparative Analysis of Two Time Periods

**DOI:** 10.1007/s12098-024-05164-4

**Published:** 2024-06-05

**Authors:** Sae Yun Kim, Jeongmin Shin, Moon Yeon Oh, Young-Ah Youn

**Affiliations:** Department of Pediatrics, College of Medicine, Seoul St. Mary’s Hospital, The Catholic University of Korea, 222 Banpo-daero, Seocho-gu, Seoul, 06591 Republic of Korea

**Keywords:** Proactive perinatal care, Minimal handling, Periviable preterm infant, Mortality

## Abstract

**Objectives:**

To investigate the impact of proactive perinatal care on periviable preterm infants before and after its implementation.

**Methods:**

This retrospective cohort study was conducted over a period of 10 y, from 2013 to 2019, referred to as Phase I, and from 2020 to 2022, referred to as Phase II. A total of 162 eligible infants born between 22^0/7^ and 25^6/7^ wk of gestation were included in this analysis.

**Results:**

A total of 125 infants were born in phase I, and 37 infants in phase II received proactive care with minimal handling. The mortality decreased from 54.4% to 24.3% (*P* = 0.001). The composite outcomes of severe brain injury or death, sepsis or death and necrotizing enterocolitis or death were also improved with proactive care. Gestational age [adjusted odds ratio (aOR) 0.900; 95% confidence interval (CI), 0.836–0.970], air leak syndrome (aOR 4.958; 95% CI, 1.681–14.624), massive pulmonary hemorrhage (aOR 4.944; 95% CI, 2.055–11.893), and birth in phase II (aOR 0.324; 95% CI, 0.115–0.912) were independently associated with mortality.

**Conclusions:**

The implementation of proactive care with minimal handling resulted in an increased survival rate and a reduction in the combined morbidities between the two time periods. The provision of proactive perinatal care with minimal handling is crucial for improving both the survival rates and clinical outcomes of these vulnerable infants.

**Supplementary Information:**

The online version contains supplementary material available at 10.1007/s12098-024-05164-4.

## Introduction

Periviability is defined as the stage of fetal maturity with a marginal chance of extrauterine survival. A recent executive summary of proceedings from a joint workshop defined periviable birth as delivery occurring from 20^0/7^ wk to 25^6/7^ wk of gestation [1]. However, because most neonates born at less than 22^0/7^ wk of gestation are virtually nonviable, periviable preterm infants (PPIs) were referred to as those born between 22^0/7^ and 25^6/7^ wk of gestation. These infants are at the border of viability, face significant challenges due to their immature physiological system and cannot survive without life-sustaining interventions immediately after delivery.

A recent recommendation from expert committees is against the routine provision of neonatal resuscitation for PPIs, unless they are considered potentially viable based on individual circumstances [2]. The decision to initiate or withhold treatment of PPI-mother dyads is a matter of debate [3] because the choice of treatment could lead to large differences in neonatal mortality and morbidities [4]. Most centers apply a selective approach depending on the views and experiences of the medical community, the individual physician, and the parents. Some neonatal intensive care units (NICUs) are offering treatment to the PPIs, whereas other NICUs are now withholding treatment [5]. This contrast is highlighted by Rysavy et al. in which interquartile ranges (IQRs) for active treatment of infants born at 22 wk of gestation were 7.7% to 100% within 24 hospitals in the United States [6].

Because proactive care for PPIs also varies from center to center, there are wide variations in the survival rate of these infants. The provision of proactive perinatal care is crucial for improving survival rates and clinical outcomes. Therefore, the purpose of this study was to investigate the direct and indirect impacts of proactive perinatal care for PPIs, comparing before and after its implementation.

## Material and Methods

This retrospective cohort study was performed at the Catholic University of Korea, Seoul St. Mary's Hospital, which is a tertiary referral university hospital with a level IV NICU. All neonates who were born between 22^0/7^ and 25^6/7^ wk of gestation and admitted to the NICU between 2013 and 2022 were eligible for the study. Infants who were born outside the hospital and transferred to this NICU, who had life-threatening congenital anomalies or hydrops fetalis, who died before 12 h after birth, or who were transferred to another hospital before postmenstrual age 36^0/7^ wk were excluded from the study. The infants were classified into two historical cohorts according to the protocols used: from January 1st, 2013, to December 31st, 2019, as phase I and from January 1st, 2020, to December 31st, 2022, as phase II. The multidisciplinary standardized protocol for proactive perinatal care was implemented in phase II.

The medical records of the infants and their mothers were reviewed. Maternal characteristics included age and mode of delivery. Maternal hypertension included gestational hypertension, pregnancy-induced hypertension, and chronic hypertension. Maternal diabetes mellitus (DM) included both gestational and overt DM. Histological chorioamnionitis was defined as described by Yoon et al. [7]. Premature rupture of membranes was defined as rupture before delivery if it lasted for > 24 h. Antenatal corticosteroid (ACS) was defined as at least one dose of ACS administered to the mother before delivery. Neonatal characteristics were classified as conditions at birth, immediate neonatal outcomes, and neonatal outcomes before discharge. The gestational age (GA) at birth was calculated according to the last menstrual period or ultrasound during the first trimester. Small for gestational age (SGA) was defined by birth weight below the 10th percentile for GA and sex [8]. Apgar scores, resuscitations in the delivery room (DR) and initial body temperature at the NICU were also collected. The bronchopulmonary dysplasia (BPD) definition was adopted from the National Institute of Child Health and Human Development (NICHD) consensus on BPD severity [9]. Sepsis was determined based on positive blood culture results. Severe brain injury was defined as intraventricular hemorrhage grade 3 or 4 on cranial ultrasonography [10] and/or periventricular leukomalacia on cranial images. Necrotizing enterocolitis (NEC) was defined as stage II or higher [11]. The composite outcome of ‘NEC or death’ was defined as death until Day 120 of life. Severe retinopathy of prematurity was defined as stage 3 or higher and/or requiring treatment [12, 13]. Most in-hospital morbidities were analyzed as both single and composite outcomes with death.

The new protocol emphasizes intensive prenatal care for high-risk mothers, proactive neonatal resuscitation starting at the DR, and minimal handling to protect the infant's brain after admission to the NICU. The primary objective of proactive perinatal care in authors’ unit was to improve the survival rate of PPIs, with the secondary goal of reducing the incidence of combined major morbidities. The protocols used during phase II are detailed in the supplementary material.

Statistical analysis was performed using SPSS version 23 (IBM Corp, Armonk, New York). Continuous variables are summarized as medians with IQRs, and dichotomous variables are presented as frequencies and percentages. Differences between groups were assessed using the Mann–Whitney U test for continuous variables and Fisher’s exact test for categorical variables. Factors with *P* < 0.05 in the univariate analysis were included in the multiple logistic regression analysis to identify independent risk factors affecting mortality. The authors used forward selection of variables in the logistic regression model and determined adjusted odds ratios (ORs) and 95% confidence intervals (CIs). Variance inflation factors were obtained to examine multicollinearity among the variables. Additionally, GA at birth and birth weight are correlated with each other; therefore, GA at birth was selected only as a confounding factor for adjustment (Pearson correlation coefficient = 0.529, *P* < 0.001). All tests were 2-tailed, and *P* < 0.05 was considered significant.

## Results

**Fig. 1:**
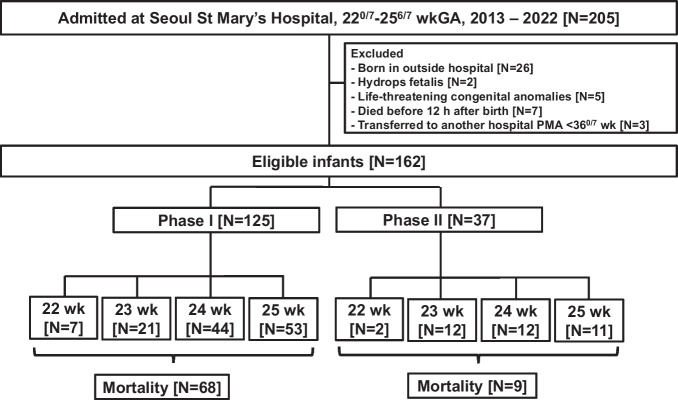
Flow chart of the study population. *GA* Gestational age, *PMA* Postmenstrual age, *wkGA* Weeks of gestational age

Over the 10-y period, a total of 205 infants born between 22^0/7^ and 25^6/7^ wk of gestation were admitted. After excluding 43 infants, the study included 162 eligible infants. During the study period, there were 9 infants born at 22 wk of gestation, 33 infants born at 23 wk of gestation, 56 infants born at 24 wk of gestation, and 64 infants born at 25 wk of gestation. Among the included infants, 125 were born in phase I, while 37 infants were born in phase II and received proactive perinatal care (Fig. 1).

**Fig. 2:**
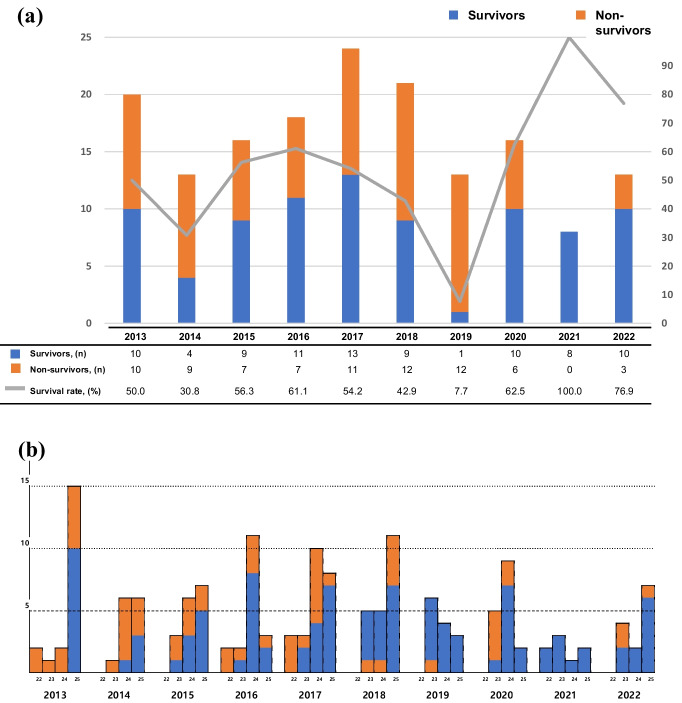
Infants born during the ten years. **a** Total number of periviable preterm infants born and survival rate for ten years. **b** Periviable preterm infants born for ten years by gestational week at birth

Overall very low birth weight (VLBW) infant mortality for 10 y was 17.2% (121/702), which decreased from 20.5% (107/522) in phase I to 7.8% (14/180) in phase II (Supplementary Table S1). The mortality of PPIs tended to decrease from 50% in 2013 to 23.1% in 2022 (Fig. 2). The initial body temperature tended to decrease toward 36 °C as authors transitioned from phase I to phase II (*P* = 0.001). The median Apgar score at 5 min tended to vary without serial changes (*P* = 0.025). Additionally, the incidence of most neonatal outcomes, the trend analysis revealed statistical significance without serial change (*P* = 0.026, *P* = 0.004, and *P* = 0.012 for severe BPD or death, severe brain injury or death, and sepsis or death, respectively). However, severe brain injury or death significantly decreased from 90% in 2013 to 46.2% in 2022. The incidence of sepsis or death significantly decreased from 70% in 2013 to 23.1% in 2022 (Table 1).

**Table 1:** Ten year trend in infant/maternal baseline clinical and demographic characteristics

	**Phase I**							**Phase II**				
	**2013**	**2014**	**2015**	**2016**	**2017**	**2018**	**2019**	**2020**	**2021**	**2022**	**Total**	***P***
No. of survivors/Total no. of births	10/20	4/13	9/16	11/18	13/24	9/21	1/13	10/16	8/8	10/13	85/162	-
Mortality	10 (50.0%)	9 (69.2%)	7 (43.8%)	7 (38.9%)	11 (45.8%)	12 (57.1%)	12 (57.1%)	6 (37.5%)	0 (0.0%)	3 (23.1%)	77 (47.5%)	0.083
Maternal age^*^	34 (8)	31.5 (7)	34.5 (4)	33.0 (8)	31.0 (5)	34.0 (4)	36.0 (7)	34.0 (5)	36.0 (11)	33.0 (5)	34 (6)	0.132
Assisted pregnancy	1 (5.0%)	1 (7.7%)	4 (25.0%)	5 (27.8%)	7 (29.2%)	10 (47.6%)	6 (46.2%)	8 (50.0%)	3 (37.5%)	2 (15.4%)	47 (29.0%)	0.008
Multiple gestation	9 (45.0%)	2 (15.4%)	6 (37.5%)	6 (33.3%)	10 (41.7%)	10 (47.6%)	4 (30.8%)	6 (37.5%)	2 (25.0%)	4 (30.8%)	59 (36.4%)	0.792
Gestational age^*^, wk	25^2/7^ (0^6/7^)	24^6/7^ (1^0/7^)	24^5/7^ (1^3/7^)	24^4/7^ (0^6/7^)	24^2/7^ (1^2/7^)	25^0/7^ (1^6/7^)	24^0/7^ (1^2/7^)	24^5/7^ (1^1/7^)	23^5/7^ (2^2/7^)	25^0/7^ (2^1/7^)	24^4/7^ (1^4/7^)	0.113
Birth weight^*^, g	765 (270)	766 (294)	649 (140)	673 (142)	660 (255)	600 (247)	742.5 (302)	653.5 (196)	594 (298)	713 (295)	688 (224)	0.073
Male sex	11 (55.0%)	8 (61.5%)	5 (31.3%)	8 (44.8%)	13 (54.2%)	11 (52.4%)	7 (53.8%)	10 (62.5%)	5 (62.5%)	6 (46.2%)	84 (51.9%)	0.682
BT at admission^*^, °C	35.9 (1)	35.85 (1)	35.25 (2)	35.5 (1)	35.8 (1)	36.2 (1)	35.9 (1)	36.25 (0)	36.5 (1)	36.05 (1)	36.0 (1)	0.001
5 min AS^*^	6 (2)	4 (3)	4 (4)	5 (2)	4 (3)	3 (3)	2.5 (3)	4 (3)	5 (2)	5 (4)	4 (4)	0.025
Treated PDA	6/20 (30.0%)	2/13 (15.4%)	5/16 (31.3%)	7/16 (43.8%)	8/22 (36.4%)	10/18 (55.6%)	4/11 (36.4%)	5/15 (33.3%)	4/7 (57.1%)	5/9 (55.6%)	56/147 (38.1%)	0.054
Severe BPD or death	13 (65.0%)	12 (92.3%)	12 (75.0%)	17 (94.0%)	22 (91.7%)	21 (100.0%)	13 (100.0%)	16 (100.0%)	6 (75.0%)	11 (84.6%)	143 (88.3%)	0.026
Severe brain injury or death	18 (90.0%)	12 (92.3%)	11 (68.8%)	11 (61.1%)	23 (95.8%)	17 (81.0%)	12 (92.3%)	7 (43.8%)	6 (75.0%)	6 (46.2%)	123 (75.9%)	0.004
Sepsis or death	14 (70.0%)	12 (92.3%)	11 (68.8%)	14 (77.8%)	17 (70.8%)	15 (71.4%)	12 (92.3%)	10 (62.5%)	5 (62.5%)	3 (23.1%)	113 (69.8%)	0.012
NEC or death	10 (50.0%)	10 (76.9%)	8 (50.0%)	10 (55.6%)	12 (50.0%)	12 (57.1%)	12 (92.3%)	8 (50.0%)	1 (12.5%)	3 (23.1%)	86 (53.1%)	0.090
Severe ROP	3/10 (30.0%)	2/4 (50.0%)	3/11 (27.3%)	6/11 (54.5%)	7/14 (50.0%)	8/9 (88.9%)	0/3 (0.0%)	2/10 (20.0%)	4/8 (50.0%)	5/10 (50.0%)	40/90 (44.4%)	0.637

Values are presented as median with interquartile range for continuous variables (denoted with ^*^) or frequencies with percentages for categorical variables, as appropriate. *P* value was calculated through χ^2^ test for trend for categorical variables and one-way ANOVA test comparing from 2013 to 2022 for continuous variables

*AS* Apgar score, *BPD* Bronchopulmonary dysplasia, *BT* Body temperature, *NEC* Necrotizing enterocolitis, *PDA* Patent ductus arteriosus, *ROP* Retinopathy of prematurity

The proportion of infants whose mothers were injected with ACS was 76.8% in phase I and 97.3% in phase II (*P* = 0.003). The GA at birth and birth weight were not different between the groups. The proportion of SGA infants decreased from 15.2% in phase I to 2.7% in phase II (*P* = 0.047). The proportions of infants who required intubation and/or were administered epinephrine at birth decreased from 99.2% in phase I to 91.9% in phase II (*P* = 0.038) and from 20.8% in phase I to 5.4% in phase II (*P* = 0.028), respectively. The median initial body temperature of the infants was 35.8 °C in phase I and 36.2 °C in phase II (*P* < 0.001). The differences in the severe BPD or death, increasing from 88.0% in phase I to 89.2% in phase II, did not reach statistical significance. The proportion of patients who experienced severe brain injury or death improved from 83.2% in phase I to 51.4% in phase II (*P* < 0.001). The incidence of sepsis or death decreased significantly, at 76.0% in phase I and 48.6% in phase II (*P* = 0.001). The incidence of NEC or death decreased from 59.2% in phase I to 32.4% in phase II (*P* = 0.005). The mortality before NICU discharge significantly decreased from 54.4% in phase I to 24.3% in phase II (*P* = 0.001).

Fifty-seven infants discharged from the NICU were alive in phase I, and 28 infants were alive in phase II. The median length of stay in the NICU became longer in phase II than in phase I (145 d and 123 d, respectively) (*P* = 0.001). The median body weight at discharge was greater in phase II than in phase I (4201 g and 3045 g, respectively) (*P* < 0.001) (Table 2).

**Table 2:** Comparison between two time periods

	**Phase I (N = 125)**	**Phase II (N = 37)**	**OR (95% CI)**	***P***
***Maternal characteristics***				
Maternal age^*^, years	34 (6)	34 (5)		0.214
Assisted pregnancy	34 (27.2%)	13 (35.1%)	1.450 (0.664, 3.167)	0.410
Diabetes mellitus	6 (4.8%)	2 (5.4%)	1.133 (0.219, 5.866)	> 0.999
Hypertension	11 (8.8%)	5 (13.5%)	1.619 (0.524, 5.0)	0.366
Oligohydramnios	26 (20.8%)	11 (29.7%)	1.611 (0.705, 3.682)	0.375
HCAM	69 (55.2%)	18 (48.6%)	0.769 (0.369, 1.603)	0.574
PROM > 24 h	47 (37.6%)	14 (37.8%)	1.010 (0.474, 2.153)	> 0.999
Antenatal steroid, any	96 (76.8%)	36 (97.3%)	10.875 (1.428, 82.798)	0.003
Cesarean delivery	99 (79.2%)	32 (86.5%)	1.681 (0.596, 4.740)	0.475
Multiple gestation	47 (37.6%)	12 (32.4%)	0.797 (0.366, 1.734)	0.698
***Neonatal characteristics at birth***				
Gestational age, at birth^*^, wk	24^5/7^ (1^3/7^)	24^4/7^ (1^6/7^)	-	0.541
Birth weight^*^, g	696.0 (219)	635.5 (241)	-	0.717
Birth weight^*^, z-score	0.04 (1.40)	0.235 (1.128)	-	0.941
Male sex	63 (50.4%)	21 (56.8%)	1.292 (0.617, 2.704)	0.577
Small for gestational age (birth weight < 10 percentile)	19 (15.2%)	1 (2.7%)	0.155 (0.020, 1.199)	0.047
Body temperature at admission^*^, °C	35.8 (1)	36.2 (0)	-	< 0.001
5 min Apgar score^*^	4 (4)	4.5 (3)	-	0.083
***Neonatal resuscitation at birth***				
Requiring intubation	124 (99.2%)	34 (91.9%)	0.091 (0.009, 0.907)	0.038
Requiring compression	26 (20.8%)	3 (8.1%)	0.336 (0.096, 1.181)	0.090
Requiring epinephrine	26 (20.8%)	2 (5.4%)	0.218 (0.049, 0.964)	0.028
***Neonatal outcomes immediately after admission***				
Air leak syndrome	28 (22.4%)	3 (8.1%)	0.306 (0.087, 1.070)	0.059
Massive pulmonary hemorrhage	45 (36.0%)	7 (18.9%)	0.415 (0.169, 1.020)	0.070
Surfactant administration	125 (100.0%)	37 (100.0%)		
Prophylactic surfactant administration	117 (90.0%)	25 (92.6%)	2.285 (0.700, 7.465)	1.000
***NICU outcomes before discharge***				
Treated PDA	42/116 (36.2%)	14/31 (45.2%)	1.451 (0.650, 3.237)	0.408
Severe BPD or death	110 (88.0%)	33 (89.2%)	1.125 (0.349, 3.623)	> 0.999
Severe brain injury	69/103 (67.0%)	12/32 (37.5%)	0.296 (0.130, 0.675)	0.003
Severe brain injury or death	104 (83.2%)	19 (51.4%)	0.213 (0.096, 0.473)	< 0.001
Sepsis	38 (30.4%)	11 (29.7%)	0.969 (0.435, 2.159)	> 0.999
Sepsis or death	95 (76.0%)	18 (48.6%)	0.299 (0.139, 0.642)	0.001
NEC ≥ 2	21 (16.8%)	3 (8.1%)	0.437 (0.123, 1.556)	0.291
NEC ≥ 2 or death	74 (59.2%)	12 (32.4%)	0.331 (0.152, 0.718)	0.005
Severe ROP	29/62 (46.8%)	11/28 (39.3%)	0.736 (0.297, 1.825)	0.647
Mortality	68 (54.4%)	9 (24.3%)	0.269 (0.118, 0.618)	0.001
No. of survivors/Total no. of births				
22–23 wkGA	6/28 (21.4%)	8/14 (57.1%)		
24–25 wkGA	51/97 (52.6%)	20/23 (87.0%)		
***Analyses among survivors***				
No. of survivors	57	28		
Length of stay^*^, days	123 (43)	145 (44)	-	< 0.001
Weight at discharge^*^, g	3045 (1095)	4201 (1240)	-	< 0.001
z-score of weight at discharge^*^	-1.13 (1.42)	-1.30 (0.88)	-	> 0.999

Values are presented as median with interquartile range for continuous variables (denoted with ^*^) or frequencies with percentages for categorical variables, as appropriate. *P* value for Fisher’s exact test (categorical measures) and Mann–Whitney U test (continuous measures) comparing two periods

*BPD* Bronchopulmonary dysplasia, *CI* Confidence interval, *HCAM* Historical chorioamnionitis, *NEC* Necrotizing enterocolitis, *OR* Odds ratios, *PDA* Patent ductus arteriosus, *PROM* Premature rupture of membrane, *ROP* Retinopathy of prematurity, *wkGA* Weeks of gestation

The following parameters were included in the multivariate logistic regression model and were significantly different between survivors and non-survivors in terms of maternal characteristics, neonatal characteristics at birth, or neonatal conditions immediately after birth: GA at birth, initial body temperature at admission, 5-min Apgar score, air leak syndrome, massive pulmonary hemorrhage, and birth in phase II. GA at birth [adjusted odds ratio (aOR), 0.900; 95% confidence interval (CI), 0.836–0.970; *P* = 0.006], air leak syndrome (aOR 4.958; 95% CI, 1.681–14.624, *P* = 0.004), massive pulmonary hemorrhage (aOR 4.944; 95% CI, 2.055–11.893; *P* < 0.001), and birth in phase II (aOR, 0.324; 95% CI, 0.115–0.912; *P* = 0.033) were independently associated with the mortality of PPIs (Table 3).

**Table 3:** Factors associated with mortality

	**Survivors(N = 85)**	**Non-survivors** **(N = 77)**	**OR (95% CI)**	***P***	**aOR (95% CI)**	***P'***
**Maternal characteristics**						
Maternal age^*^, years	33.5 (7)	35 (6)	-	0.789		
Assisted pregnancy	22 (25.9%)	25/76 (32.5%)	1.377 (0.697, 2.719)	0.388		
Diabetes mellitus	6 (7.1%)	2 (2.6%)	0.351 (0.069, 1.794)	0.282		
Hypertension	12 (14.1%)	4 (5.2%)	0.333 (0.103, 1.082)	0.068		
Oligohydramnios	16 (18.8%)	21 (27.3%)	1.617 (0.772, 3.389)	0.259		
HCAM	51 (60.0%)	36 (46.8%)	0.585 (0.314, 1.092)	0.115		
PROM > 24 h	33 (38.8%)	28 (36.4%)	0.900 (0.476, 1.703)	0.871		
Antenatal steroid, any	72 (84.7%)	60 (77.9%)	0.637 (0.287, 1.417)	0.314		
Cesarean delivery	70 (82.4%)	61 (79.2%)	0.817 (0.373, 1.789)	0.691		
Multiple gestation	28 (332.9%)	31 (40.3%)	1.372 (0.722, 2.607)	0.414		
**Neonatal characteristics at birth**						
Gestational age, at birth^*^, wk	25^0/7^ (1^2/7^)	24^2/7^ (1^4/7^)	-	< 0.001	0.900 (0.836, 0.970)	0.006
Birth weight^*^, g	725.5 (211)	613 (235)	-	0.001		
Birth weight, z-score^*^	0.32 (1.098)	-0.12 (1.31)	-	0.052		
Male sex	44 (51.8%)	40 (51.9%)	1.007 (0.543, 1.867)	> 0.999		
SGA < 10 percentile	7 (8.2%)	13 (16.9%)	2.263 (0.852, 6.010)	0.150		
Body temperature at admission^*^, °C	36 (1)	35.8 (1)	-	0.022	0.756 (0.460, 1.241)	0.269
5 min Apgar score^*^	5 (2)	3 (3)	-	< 0.001	0.839 (0.656, 1.072)	0.160
**Neonatal resuscitation at birth**						
Requiring intubation	81 (95.3%)	77 (100.0%)	-	0.122		
Requiring compression	12 (14.1%)	17 (22.1%)	1.724 (0.764, 3.890)	0.221		
Requiring epinephrine	12 (14.1%)	16 (20.8%)	1.596 (0.701, 3.630)	0.302		
**Neonatal conditions immediately after birth**						
Air leak syndrome	6 (7.1%)	25 (32.5%)	6.330 (2.430, 16.488)	< 0.001	4.958 (1.681, 14.624)	0.004
Massive pulmonary hemorrhage	12 (14.1%)	40 (51.9%)	6.577 (3.086, 14.016)	< 0.001	4.944 (2.055, 11.893)	< 0.001
Surfactant administration	85 (100.0%)	77 (100.0%)	-			
Prophylactic	80 (93.0%)	73 (90.1%)	1.461 (0.484, 4.3412)	0.582		
***Birth during phase I***	57/85 (67.1%)	68/77 (88.3%)				
***Birth during phase II***	28/85 (32.9%)	9/77 (11.7%)	0.269 (0.111, 0.618)	0.001	0.324 (0.115, 0.912)	0.033

Values are presented as median with interquartile range for continuous variables (denoted with ^*^) or frequencies with percentages for categorical variables, as appropriate. OR and *P* value for Fisher’s exact test (categorical measures) and Mann–Whitney U test (continuous measures) comparing two groups. aOR and *P*' value are calculated from binary logistic regression adjusted with co-varying factors (GA, BT, 5AS, air leak, MPH, phase I/phase II)

*aOR* Adjusted odds ratios, *5AS* 5 min Apgar score, *BT* Body temperature, *CI* Confidence interval, *GA* Gestational age, *HCAM* Historical chorioamnionitis, *MPH* Massive pulmonary hemorrhage, *OR* Odds ratio, *PROM* Premature rupture of membrane, *ROP* Retinopathy of prematurity, *SGA* Small for gestational age

## Discussion

In the present study, authors observed that the implementation of proactive perinatal care, beginning from intensive prenatal care for high-risk mothers, continuing proactive neonatal resuscitation starting at the DR, and minimizing handling by reducing unnecessary sampling and tests in NICU, led to improvements in survival rates as well as reductions in neonatal morbidities among PPIs. First, the mortality rate decreased significantly, dropping from 54.4% to 24.3% after the implementation of proactive perinatal care for PPIs. Proactive perinatal care emerged as an independent factor contributing to this reduction in the mortality rate. Second, the incidence of neonatal morbidities or death, especially severe brain injury or death, significantly decreased, which could be considered an indicator of improved long-term neurological outcomes.

The survival of PPIs born at 22^0/7^ to 24^6/7^ wk of gestation increased from 30% (424/1391) to 36% (487/1348) from 2000–2003 to 2008–2011 [14]. However, these outcomes differ depending on the treatment policy. PPIs may receive comfort-focused or survival-focused care following delivery. The goal of comfort care is to provide comprehensive health care services to avoid painful stimuli or maternal-infant separation to mother-infant dyads with life-limiting conditions [15]. Meanwhile, for survival care, health care professionals provide maximal resuscitation to PPIs. These variations in neonatal care have a substantial impact on the survival and morbidities of PPIs. Clearly, more active perinatal care has resulted in higher survival rates among PPIs. In Western Europe, routine provision of proactive treatment is not standard care. The reported survival rates were only 0% in the EPICURE study in England [16] and 2% in the EPIPAGE-2 study in France [17] for infants born at 22 wk of gestation. In contrast, with proactive treatment in highly specialized centers, survival rates above 50% can be routinely achieved [18]. Furthermore, several studies in which proactive treatment was provided to all mother–infant dyads at risk for periviable delivery have provided evidence for these significantly improved statistics [18–22].

It is worth to note that the survival rate in present study increased dramatically after the provision of proactive treatment—75.7% in phase II—which was even greater than that in the US or Sweden. Logistic regression analyses revealed that initial conditions such as GA at birth, air leak syndrome, and massive pulmonary hemorrhage were independently associated with mortality of PPIs, which is consistent with the findings of previous studies [23, 24]. Each additional week for GA resulted in a 0.9-fold decrease in the risk of mortality. Moreover, neonatal conditions immediately after birth were associated with a greater mortality risk: 4.958 times and 4.944 times greater for patients with air leak syndrome and massive pulmonary hemorrhage, respectively. Furthermore, birth during phase II decreased the risk of mortality by 0.324 times. These findings proved again that proactive perinatal care for PPIs confers survival benefits.

Higher survival rates were achieved in the phase II than that of recent results based on nationwide South Korea’s registry [25], and the survival rate in present study reached as high as that of US and Sweden [19, 20]. The special characteristic of the present proactive perinatal care protocol is that three aspects of effort were made together: intensive prenatal care for high-risk mothers, proactive resuscitation for PPIs immediately after birth, and minimal handling with maximal observation after admission to the NICU. Prenatally, the administration of ACS significantly increased during phase II. In a study from the NICHD, Neonatal Research Network, high mortality was reported for PPIs without maternal ACS administration [6]. Since the experienced attending neonatologist efficiently led the neonatal resuscitation before the baby's condition deteriorated, the initial body temperature at admission also significantly increased in phase II, and the proportion of neonates who needed epinephrine during resuscitation significantly decreased with the new protocols. These findings supported that authors’ center’s multidisciplinary approach unquestionably contributed to the improved survival of PPIs and a reduction in overall morbidities.

A recent study reported that infants who received active perinatal care had no or mild long-term neurodevelopmental impairment [14, 18, 26]. Minimal handling, which is implemented for neuroprotective purposes, reduces stress and can enhance the potential for neuroplasticity in PPIs, thereby promoting healthier brain development. Although the authors did not analyze long-term outcomes, morbidities other than BPD decreased in phase II. In particular, severe brain injury or death significantly decreased from 83.2% to 51.4% with time, which could be considered an indicator of improved long-term neurological outcomes.

The main strength of the current study is uniqueness of the protocol, as it considered intensive obstetric care, proactive perinatal care and minimal handling together. However, this study had several limitations. First, this was a retrospective study design, which might be unable to fully confirm the examined relationships. Second, the study group had a relatively small sample size. Third, there was a consistent decreasing trend in the number of births in authors’ center, especially after the onset of Coronavirus disease 2019 pandemic, when the total number of births decreased in South Korea.

## Conclusions

The present study provides clear evidence of an independent association between proactive perinatal care and a lower mortality rate. This finding highlights the significant impact of proactive perinatal care on improving the survival and clinical outcomes for PPIs. Furthermore, the provision of active care has a positive effect on the long-term neurodevelopment of these infants. Future research is warranted to determine the associations between proactive management and survival without severe disability in PPIs to improve long-term quality of life, especially neurologic outcomes.

## Supplementary Information

Below is the link to the electronic supplementary material.

Supplementary file1 (DOCX 27 KB)
