# Supplementary material for: Enhanced Survival of 22–25 Week Preterm Infants After Proactive Care Implementation: A Comparative Analysis of Two Time Periods
Source: Indian J Pediatr. 2024 Jun 5;92(8):844–52. doi: 10.1007/s12098-024-05164-4 (PMC12279888; doi:10.1007/s12098-024-05164-4)
Supplement: Supplementary file 1 — Supplementary file1 (DOCX 27 KB) [file 12098_2024_5164_MOESM1_ESM.docx]

**Supplementary material**

**Protocol of Proactive Perinatal Management**

1. **Obstetrical Care Considerations**

Intrapartum continuous fetal heart rate monitoring is routinely performed. If labor is threatening, following prenatal management protocols are implemented by obstetrical staff:

(1) Provision of antenatal steroids at 22^0/7^ wk gestation if labor is imminent, for steroid effect

(2) Provision of tocolytics, as indicated, if labor is imminent, to enhance latency

(3) Maternal antibiotics as indicated if signs of chorioamnionitis, and peripartum antibiotic prophylaxis as per routine for preterm deliveries

(4) Magnesium sulfate for neuroprotection according to national guidelines.

**2. Proactive Neonatal Resuscitation at Delivery Room**

When the birth of periviable preterm infants is approaching, following postnatal management protocols are implemented by attending neonatologist:

(1) DR prenatal set up is required; increasing the number of physicians who participated in the resuscitation (≥4 physicians, more than two attending neonatologists for one baby) and having more qualified personnel participating in the resuscitation. Highly experienced neonatologist and clinical nurse practitioner who are specialized in preterm infant care for more than 7 y attend all deliveries at these gestations.

(2) DR preparation for avoiding hypothermia at NICU admission; plastic bags, caps, and radiant warmer were used for the neonates, and the temperature of the DR or the operating room are set above 23.0°C.

(3) Immediate endotracheal intubation, followed by standard dose of surfactant replacement therapy with gentle positive pressure ventilation is recommended.

(4) Waiting 7 to 10 min for oxyhemoglobin saturations to rise to 90% with monitoring for heart rates.

(5) Transferring the neonate over the shortest distance from the delivery room to the NICU as soon as possible.

**3. After NICU admission; temperature and humidification for closed incubators**

(1) After admission to the NICU, initial care is initiated once the body temperature has been raised to above 36°C, which includes weighing. However, in cases of hypoglycemia, priority is given to establishing an intravenous line.

(2) If the body temperature is below 35°C, caution is exercised to avoid rapid warming, with a maximum rate of 1°C per hour. Rapid warming can potentially lead to increased insensible water losses, heightened risk of intraventricular hemorrhage, and elevated oxygen consumption.

(3) For the first day to 72 h, the humidity: 80-90%

The initial 72-h incubator humidity is set above 90% on the first day, gradually decreasing to 75% by 7 d of age. By 14 d of age, humidity level is maintained at around 60%. This protocol is essential due to various medical conditions, such as advancement to non-invasive ventilatory support or phototherapy, which can increase insensible water loss.

**4. Minimal handling with maximal observation**

(1) Implementing minimal handling and thorough monitoring of fluid status, including calculation of insensible losses, alongside appropriate humidification settings.

(2) Minimizing invasive procedures such as blood sampling with maximal careful observation especially within 14 d

(3) Hydrocortisone use: Preterm infants born at 22-25 wk of gestation often exhibit impaired cortisol production due to the reliance on progesterone supplied by the placenta for cortisol synthesis during fetal life [1]. Therefore, the early and appropriate use of hydrocortisone, typically at a dose of 1-2 mg/kg, for adrenal insufficiency as a physiological glucocorticoid dose, can significantly contribute to circulatory support.

(4) Promoting collaborative efforts between nursing and medical staff, emphasizing active communication and adopting a clustered approach to handling periviable preterm infants, thereby reducing the overall number of procedures.

**References**

1. Masumoto K, Kusuda S. Hemodynamic support of the micropreemie: should hydrocortisone never be left out? Semin Fetal Neonatal Med. 2021; https://doi.org/10.1016/j.siny.2021.101222.

**Supplementary Table S1.** Mortality of periviable preterm infants and VLBW infants for 10 y

|  |  | **Phase I** | | | | | | | **Phase II** | | |  |
| --- | --- | --- | --- | --- | --- | --- | --- | --- | --- | --- | --- | --- |
|  |  | **2013** | **2014** | **2015** | **2016** | **2017** | **2018** | **2019** | **2020** | **2021** | **2022** | **Total** |
| **VLBW** | **No. of survivors/ Total no. of births** | 65/85 | 54/76 | 81/89 | 58/67 | 59/79 | 57/70 | 41/56 | 43/53 | 58/59 | 65/68 | 581/702 |
|  | **No. of infants, died** | 20 | 22 | 8 | 9 | 20 | 13 | 15 | 10 | 1 | 3 | 121 |
|  | **Mortality (%)** | 23.5 | 28.9 | 9.0 | 13.4 | 25.3 | 18.6 | 26.8 | 18.9 | 1.7 | 4.4 | 17.2 |
|  |  | **Phase I mortality 20.5% (107/522)** | | | | | | | **Phase II mortality 7.8% (14/180)** | | |  |
| **22-25**  **wk GA** | **No. of survivors/ Total no. of births** | 10/20 | 4/13 | 9/16 | 11/18 | 13/24 | 9/21 | 1/13 | 10/16 | 8/8 | 10/13 | 85/162 |
|  | **22 wkGA** | 0/2 | 0 | 0 | 0/2 | 0/3 | 0 | 0 | 0 | 2/2 | 0 | 2/9 |
|  | **23 wkGA** | 0/1 | 0/1 | 1/3 | 1/2 | 2/3 | 1/5 | 1/6 | 1/5 | 3/3 | 2/4 | 12/33 |
|  | **24 wkGA** | 0/2 | 1/6 | 3/6 | 8/11 | 4/10 | 1/5 | 0/4 | 7/9 | 1/1 | 2/2 | 27/56 |
|  | **25 wkGA** | 10/15 | 3/6 | 5/7 | 2/3 | 7/8 | 7/11 | 0/3 | 2/2 | 2/2 | 6/7 | 44/64 |
|  |  | **Phase I mortality 54.4% (68/125)** | | | | | | | **Phase II mortality 24.4% (9/37)** | | |  |

*VLBW* Very low birth weight; *wkGA* Weeks of gestation
